# Can N Fertilizer Addition Affect N_2_O Isotopocule Signatures for Soil N_2_O Source Partitioning?

**DOI:** 10.3390/ijerph18095024

**Published:** 2021-05-10

**Authors:** Peiyi Zhang, Teng Wen, Yangmei Hu, Jinbo Zhang, Zucong Cai

**Affiliations:** 1 School of Geography Science, Nanjing Normal University, Nanjing 210023, China; wtjessy@163.com (P.Z.); myfightinglife@126.com (Y.H.); 09324@njnu.edu.cn (J.Z.); zccai_sp@163.com (Z.C.); 2 Jiangsu Center for Collaborative Innovation in Geographical Information Resource Development and Application, Nanjing Normal University, Nanjing 210023, China; 3 Key Laboratory of Virtual Geographic Environment (Nanjing Normal University), Ministry of Education, Nanjing 210023, China

**Keywords:** isotopocule, nitrous oxide, N fertilizer, soil incubation

## Abstract

Isotopocule signatures of N_2_O (δ^15^N^bulk^, δ^18^O and site preference) are useful for discerning soil N_2_O source, but sometimes, N fertilizer is needed to ensure that there is enough N_2_O flux for accurate isotopocule measurements. However, whether fertilizer affects these measurements is unknown. This study evaluated a gradient of NH_4_NO_3_ addition on N_2_O productions and isotopocule values in two acidic subtropical soils. The results showed that N_2_O production rates obviously amplified with increasing NH_4_NO_3_ (*p* < 0.01), although a lower N_2_O production rate and an increasing extent appeared in forest soil. The δ^15^N^bulk^ of N_2_O produced in forest soil was progressively enriched when more NH_4_NO_3_ was added, while becoming more depleted of agricultural soil. Moreover, the N_2_O site preference (SP) values collectively elevated with increasing NH_4_NO_3_ in both soils, indicating that N_2_O contributions changed. The increased N_2_O production in agricultural soil was predominantly due to the added NH_4_NO_3_ via autotrophic nitrification and fungal denitrification (beyond 50%), which significantly increased with added NH_4_NO_3_, whereas soil organic nitrogen contributed most to N_2_O production in forest soil, probably via heterotrophic nitrification. Lacking the characteristic SP of heterotrophic nitrification, its N_2_O contribution change cannot be accurately identified yet. Overall, N fertilizer should be applied strictly according to the field application rate or N deposition amount when using isotopocule signatures to estimate soil N_2_O processes.

## 1. Introduction

The nitrous oxide (N_2_O) emitted from soil is governed by various pathways, which often occur simultaneously in different soil micro-sites [1,2]. To adopt appropriate mitigation strategies, attribution of the source of emitted N_2_O is very important. Recently, a position-specific nitrogen (N) isotope method measuring the intramolecular distribution of ^15^N in N_2_O (site preference, SP) has served as a useful tool to source partition N_2_O in various soils [3,4,5,6]. It has clear advantages, such as the minimal disturbance of soil over the ^15^N tracing method, independence of the isotopic signature of the substrate over the traditional natural abundance (^15^N, ^18^O) method and applicability in spatial–temporal scales with a low cost [7,8,9].

However, compared with bulk ^15^N (δ^15^N^bulk^) and ^18^O (δ^18^O) measurements, obtaining highly accurate SP measurements is challenging, not only because it is indirectly determined by bulk ^15^N and α-^15^N (central position), but also because N_2_O isotopocules overlapping and ^15^N scrambling in an ion source both propagate analytical errors [5,10]. To ensure precise and robust isotopocule measurements, this method is mainly performed in soils with a high N_2_O concentration, such as agricultural and grassland soil, while it is seldom utilized in forest soil due to the relatively small N_2_O flux [3]. Only some tropical and frozen forest soils producing a significantly high N_2_O flux have been a constrained N_2_O source via this method [11,12,13]. To obtain a sufficient N_2_O quantity for isotopocule analysis, some researchers used an improved chamber technique with a molecular sieve to continuously trap N_2_O in field experiments [14], while others added N fertilizers (KNO_3_, urea, NH_4_NO_3_, etc.) to increase N_2_O flux in incubation experiments [15,16,17,18]. N fertilizer could enhance N_2_O production, but excess fertilizer might induce a priming effect, change the N_2_O efflux and bias the N_2_O source [19]. Agricultural and grassland soil is frequently applied with N fertilizer, commonly according to its actual application amount [3], but the appropriate N fertilizer application ratio in forest soil is hard to estimate. In the literature, the lowest application rate was 52.4 mg urea-N kg^−1^ (in line with the N deposition amount) in tropical lowland forest soil [15] and the highest rate was up to 1470 mg KNO_3_-N Kg^−1^ in temperate forest soil [18]. Such high application rates are comparable to that in agricultural soil, but whether they influence N_2_O isotopocule signatures and the subsequent N_2_O origin analysis has never been clearly defined. The purpose of this study was to investigate the effect of N fertilizer application on the isotopocule signatures of the N_2_O produced in forest soil and determine its potential influence on N_2_O source partition. Moreover, we selected an acidic forest soil in subtropical China that has been reported to have large N_2_O emissions [20,21], but its isotopocule values have not been reported.

Our aim was to test whether isotopocule values of soil-emitted N_2_O (δ^15^N^bulk^, δ^18^O and SP) differed and whether the contributions of N_2_O pathways changed after applying a gradient of NH_4_NO_3_. Two types of acidic soil (agricultural and forest) in a subtropical area were selected.

## 2. Materials and Methods

### 2.1. Soil Properties

The subtropical soils investigated were sampled from Jiangxi Province (27°59′N, 117°25′E), China, in May 2018, of which one was agricultural soil (with three replicates collected from three upland agricultural areas) and the other was forest soil (with three replicates). The total N_2_O emissions estimated from agronomy (maize, rice, wheat and vegetable crop) in Jiangxi Province were 0.03, 1.55, 0.01 and 1.13 Gg N yr^−^^1^, respectively [22], while the annual cumulative N_2_O flux from forest was 0.95 ± 0.21 kg N ha^−^^1^yr^−^^1^ on average [23]. The dominant vegetation in forest soil was *Pinus massoniana* and *Cunninghamia lanceolate*. The agricultural soil was upland maize soil, which was established by clearing the native forest and has been applied at about 200–300 kg N ha^−1^ per year for 10 years. The two types of soil were different in parameters associated with N_2_O turnover, i.e., soil organic C and total nitrogen. The sampled area was a typical subtropical climate region, where the mean annual precipitation and temperature were 1785 mm and 18.4 °C, respectively. The surface soils (0–20 cm) were sampled at three randomly arranged places. Each type of soil sample was sieved through a 2-millimeter screen, mixed for homogeneity and then stored at 4 °C prior to incubation.

A 50-gram subsample of each soil type was air-dried for basic physicochemical properties’ investigation (Table 1). Soil pH was analyzed in a soil:water ratio of 1:2.5 (w/v). SOC, total N and C/N ratio were all determined by a CN element analyzer (Sercon Europa EA-GSL, UK). NH_4_-N and NO_3_-N were extracted using 2 M KCl in a soil:solution ratio of 1:5 (w/v) and analyzed by a continuous-flow analyzer (SA1000, Skalar, The Netherlands).

### 2.2. Experiments to Determine Effect of NH_4_NO_3_ Addition on Isotopocule Ratios of N_2_O

For each sample, 150 g fresh soil (oven-dry basis) was packed into a 500-milliliter conical flask. The soils were applied with 0, 20, 40, 80 and 160 mg N kg^−1^ soil NH_4_NO_3_ uniformly. This gradient of NH_4_NO_3_ was set according to the rates most frequently applied in soil experiments using N_2_O isotopocules. Then soils were adjusted to 60% maximum water-holding capacity using deionized water. Each treatment had 5 replicates. All flasks were sealed by rubber stops with plastic tubes as inlet and outlet flow lines. Before incubation and after each gas sampling, the flasks with soil samples were vacuumed and filled with synthetic air (N_2_O free) three times. The soils were incubated for 48 h at 25 °C and gas samples were collected at 24 h intervals. A 5-milliliter gas sample was collected from each flask for N_2_O concentration measurements. Another 50-milliliter gas sample was collected into a pre-evacuated bottle to determine the isotopocule ratios. The δ^15^N^bulk^, δ^18^O and SP values from the gas samples were analyzed by a Delta V plus IRMS (Thermo Fisher Scientific, China), which was equipped with five cups to analyze both m/z 44, 45 and 46 of N_2_O molecules and m/z 30 and 31 of NO^+^ fragments. The scrambling factor in the ion source of this IRMS (0.085) was determined, as Röckmann [24] reported, using a series of β-labeled N_2_O. The laboratory’s N_2_O working standard was two-point, calibrated by two standards kindly offered by Dr. Reinhard Well and Dr. Anette Giesemann (Thünen Institute of Climate-Smart Agriculture, Germany). The nonlinear effect caused by various sample N_2_O amounts was corrected by a series of different standard gas mol fractions (0.3, 1, 5, 10 and 20 ppm), which were analyzed within each sample run. The δ^15^N^α^, δ^15^N^bulk^ and SP were calculated according to Equations (1) and (2) [5]. The typical analytical precision for δ^15^N^bulk^, δ^18^O and SP in this IRMS was 0.3, 0.6 and 0.9‰, respectively. The N_2_O concentrations were determined by a gas chromatograph fitted with an electron capture detector (Agilent 7890).

(1) $$\;\delta^{15}N^{bulk}=\left(\delta^{15}N^{\alpha }+\delta^{15}N^{\beta }\right)/2^{\;}$$

(2) $$SP=\delta^{15}N^{\alpha }-\delta^{15}N^{\beta }$$

### 2.3. Statistical Analysis

All statistical analyses were applied using SPSS 19. The data were checked for normality (Shapiro–Wilk test and Q–Q plot) and homogeneity of variances (Levine’s test) before statistical analysis. The N_2_O flux was log_10_ transformed to obtain a normal distribution. Then, one-way ANOVA analysis and the least significant difference (Tukey) at a level of *p* < 0.05 were used to compare the N_2_O production rates and isotopic signatures of N_2_O under different levels of fertilization.

### 2.4. N_2_O Source Partition by the Two-End-Member Mixing Approach

The two-end-member mixing model [25] was used to estimate the contributions of soil-emitted N_2_O by denitrifier denitrification/nitrifer denitrification (lower SP values) and by autotrophic nitrification/fungal denitrification (higher SP values). Therefore, four cases were considered: Case 1 is autotrophic nitrification versus nitrifier denitrification, Case 2 is autotrophic nitrification versus denitrifier denitrification, Case 3 is fungal denitrification versus nitrifier denitrification and Case 4 is fungal denitrification versus denitrifier denitrification. As the SP values in this study were not low, we excluded the simultaneous occurrence of denitrifier denitrification and nitrifier denitrification, as per Zou et al. [25]. The contribution of N_2_O (*x*) from denitrifier denitrification in Case 1 can be calculated as Equation (3). If N_2_O reduction happens, its SP will increase along a slope of 1.2 ± 0.5 [26]. The contribution of each end member can be calculated by the intersection of the reduction line and the mixing line using Equation (3). The calculations of each end member’s contribution in Cases 2–4 are calculated as in Case 1. Admittedly, this method might overestimate the contributions from the two end members in each case.

(3) $$SP_{sample}=xSP_{denitrifier\;denitrification}+\left(1-x\right)SP_{autotrophic\;nitrification}$$

## 3. Results

### 3.1. Soil Properties

The soil physical and chemical properties prior to the experiment are reported in Table 1. Both types of soil were acidic with a pH ≤ 4.8, and their dominant inorganic N was NO_3_^−^. The soil organic carbon (SOC) and the C/N ratio in forest soil were twice as high as those in agricultural soil. However, higher NH_4_^+^ and NO_3_^−^ concentrations were observed in agricultural soil, whose NO_3_^−^ (60.7 mg kg^−1^) was even over four times higher than that in forest soil (13.8 mg kg^−1^).

### 3.2. N_2_O Production Rates

The N_2_O production rates in both agricultural and forest soils obviously increased with increasing NH_4_NO_3_ addition (*p* < 0.01), while the extent of its increase in agricultural soil was much greater than that in forest soil (Table 2 and Table 3). The N_2_O production rate in agricultural soil was 3.0 μg kg^−1^ d^−1^ without NH_4_NO_3_ addition; then, it significantly elevated by about 25% when 20 mg N kg^−1^ soil N was added (*p* < 0.01) and doubled (6.0 μg kg^−1^ d^−1^) when 160 mg N kg^−1^ soil N was added (*p* < 0.01). In forest soil, the N_2_O production rate was comparatively lower and slowly growing with increasing added NH_4_NO_3_. It was 1.8 μg kg^−1^ d^−1^ without NH_4_NO_3_ addition, gradually increased to 2.0 μg kg^−1^ d^−1^ when 40 mg N kg^−1^ soil N was added and stayed the same with more NH_4_NO_3_ added.

### 3.3. Isotopocule Values of N_2_O

The δ^15^N^bulk^ of N_2_O emitted in agricultural soil generally became progressively lighter with more NH_4_NO_3_ added, while it became gradually enriched in forest soil (Table 2 and Table 3). It was -26.4‰ in agricultural soil without adding NH_4_NO_3_, then decreased to −32.2‰ when 20 mg N kg^−1^ soil N was added and further depleted to −38.1‰ after applying 160 mg N kg^−1^ soil N. Negative correlations between the NH_4_NO_3_ level and δ^15^N^bulk^ values were observed in agricultural soil (*r* = −0.759, *p* < 0.001). The δ^15^N^bulk^ of N_2_O emitted in forest soil was higher than that in agricultural soil across 0–160 mg N kg^−1^ soil levels. It was −14.6‰ without adding NH_4_NO_3_ and slowly increased to −10.7‰ with addition of 160 mg N kg^−1^ soil N. Therefore, the difference in δ^15^N^bulk^ values of N_2_O emitted between agricultural and forest soil was about 12‰ without adding NH_4_NO_3_ and increased to 28‰ when 160 mg N kg^−1^ soil N was added. Compared with the obvious change in δ^15^N^bulk^ values, δ^18^O values of N_2_O in agricultural soil only slightly fluctuated with increasing NH_4_NO_3_, while they showed a slow growth trend in forest soil.

The SP values of N_2_O emitted in agricultural soil and forest soil slightly but significantly increased with increasing NH_4_NO_3_ addition (Table 2 and Table 3). In agricultural soil, the increment was not significant with 20, 40 and 80 mg N kg^−1^ soil addition (*p* > 0.05), except the 160 mg N kg^−1^ soil addition. Compared with agricultural soil, the effect of NH_4_NO_3_ addition was more obvious in forest soil. Its SP values significantly increased when 80 mg N kg^−1^ soil was applied (*p* < 0.05). Relative to agricultural soil, the SP values of N_2_O in forest soil were generally 10‰ lower across 0–160 mg N kg^−1^ soil levels.

### 3.4. N_2_O Source Contributions

**Figure 1:**
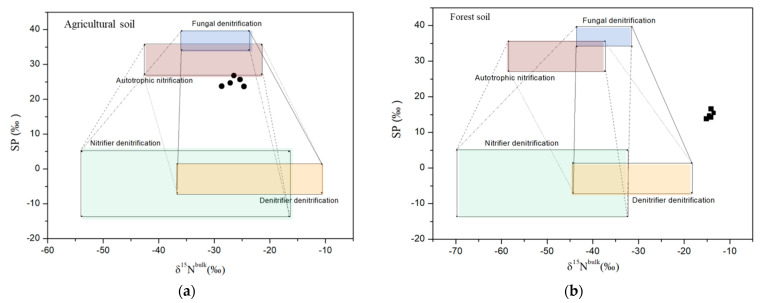
Relations between SP and δ^15^N^bulk^ of N_2_O produced in agricultural soil (**a**) and forest soil (**b**) without NH_4_NO_3_ addition. The boxes indicate the expected ranges of N_2_O produced by autotrophic nitrification, fungal denitrification, nitrifier denitrification and denitrifier denitrification. The dash lines denote the mixing zone of Case 1 (autotrophic nitrification and nitrifier denitrification are end members); solid lines denote the mixing zone of Case 2 (nitrifier denitrification and denitrifier denitrification are end members); dash dot lines denote the mixing zone of Case 3 (fungal denitrification and nitrifier denitrification are end members); short dot lines denote the mixing zone of Case 4 (fungal denitrification and denitrifier denitrification are end members). The black circles denote the N_2_O samples collected in agricultural soil and the black squares denote the N_2_O samples collected in forest soil.

The isotopocule values of N_2_O emitted in agricultural and forest soil are shown in the isotopocule map (Figure 1). All of the samples collected from agricultural soil were located between the mixing zones of the four processes, and their distributions were very close to the autotrophic nitrification and fungal denitrification processes. However, all of the samples obtained from forest soil were located outside the four processes’ mixing zone and they did not show a clear preference for each process. The two-end-member mixing results (Table 4), based on the δ^15^N^bulk^-SP map, showed that the contributions of various N_2_O processes changed when more NH_4_NO_3_ was added. Autotrophic nitrification and fungal denitrification contributed just slightly more to N_2_O emission in agricultural soil without NH_4_NO_3_ but became dominant N_2_O pathways when amounts of 40 and 80 mg N kg^−1^ soil NH_4_NO_3_ were applied. With 160 mg N kg^−1^ soil NH_4_NO_3_, their contributions modestly decreased but were still higher than those without NH_4_NO_3_ addition in some cases. In forest soil, the contributions of N_2_O processes in Cases 2 and 4 clearly changed with the increasing NH_4_NO_3_ addition.

## 4. Discussion

In this study, we have shown that N fertilizer addition led to an obvious change in the N_2_O flux, N_2_O isotopocule signatures and N_2_O source contributions in agricultural and forest soil incubation experiments.

Earlier findings showed that N fertilizer addition in soil could increase the N_2_O production rate [27,28,29]. Our results reinforced that added NH_4_NO_3_ significantly enhanced the N_2_O production rate in both agricultural and forest soils with different magnitudes. However, with the increasing N_2_O flux in agricultural and forest soil, their isotopocule signatures obviously changed with NH_4_NO_3_ addition as well. The δ^15^N^bulk^ values in the two soils were very negative but exhibited opposite trends with increasing NH_4_NO_3_. In fact, the δ^15^N^bulk^ values in both soils were within the reported isotopocule signature ranges in the literature (−67.5 to 4.2‰ in forest soil and −66.7 to 6.0‰ in agricultural soil) [3]. Soil incubation studies commonly reported skew discrimination of ^15^N because substrate diffusion is not a limiting factor and microbial N_2_O production can get close to the maximum apparent isotope effect [3]. In the agricultural soil, the sufficient NH_4_^+^and NO_3_^−^ substrates provided by NH_4_NO_3_ addition amplified such a ^15^N discrimination in N_2_O production, so its δ^15^N^bulk^ values were more depleted with the enhancing NH_4_NO_3_ level. In forest soil, our previous ^15^N tracing studies found that N_2_O was produced mainly from an organic N pool [21,30], which was not directly supplemented by added NH_4_NO_3_. Therefore, the ^15^N discrimination was reduced and the δ^15^N^bulk^ values were progressively enriched with increasing NH_4_NO_3_ when more organic N was consumed to produce N_2_O. Compared with the profound change in δ^15^N^bulk^ values, δ^18^O showed moderate changes in both soils. This is probably because the O isotopic composition of N_2_O not only depends on the substrate compounds but also on the O_2_ involved in ammonium/hydroxylamine oxidation and O exchange with H_2_O in denitrification [5,31].

It was somewhat surprising that the SP values of the produced N_2_O in the two types of soil collectively elevated with increasing NH_4_NO_3_. The enhancing SP probably indicated changing contributions from various N_2_O pathways, because SP is independent of the isotopic signatures of a substrate. The SP values of N_2_O production pathways can be divided into two groups: bacterial nitrification (average 31.4‰) and fungal denitrification (average 37‰) are specified with a higher SP, while nitrifier denitrification (average −3.8‰) and bacterial denitrifier denitrification (average −2‰) are characterized by a lower SP [8]. The high SP values (24.9~28‰) in the agricultural soil suggested that autotrophic nitrification or fungal denitrification contributed the most to N_2_O emission with or without NH_4_NO_3_ addition, but their contributions were obviously amplified when more NH_4_NO_3_ was applied (Table 4). It seemed that NH_4_NO_3_ addition would overestimate the influence of the two processes in agricultural soil. The SP values in forest soil (15~18.4‰) were relatively low, but all were located outside the mixing zone of the four processes (Figure 1). This phenomenon might occur when a large amount of N_2_O reduces to N_2_, or other N_2_O pathways whose SP values have not been illustrated as contributing the most. Since N_2_O and NO account for 80% of denitrification gas products even under very anaerobic conditions in this soil [32], it is probably heterotrophic nitrification that plays a dominant role in soil N_2_O production. Due to lacking the SP signature of heterotrophic nitrification, the two-end-member mixing model results were based on only four processes that might overestimate the impact of the denitrification processes. However, obvious contribution shifts in Cases 2 and 4 were observed after the NH_4_NO_3_ addition in forest soil.

Furthermore, we can use isotopocule and N_2_O flux data to investigate how exogenous NH_4_NO_3_ input alters N_2_O-producing processes. In the agricultural soil, the positive increase in the autotrophic nitrification and fungal denitrification was the result of NH_4_NO_3_ addition, which provided more NH_4_^+^ substrates for nitrifying bacteria and NO_3_^−^ substrates for denitrifying fungal [33]. Therefore, its N_2_O production rates exhibited a positive elevation with added NH_4_NO_3_, but its δ^15^N^bulk^ of N_2_O became more depleted. In the forest soil, the N_2_O production rate increased when 20 and 40 mg N kg^−1^ NH_4_NO_3_ were added but did not further increase when more NH_4_NO_3_ was added. This indicated that a different priming mechanism was occurring in the forest soil. Some studies reported that increased labile N can trigger carbon limitations in microbes and then stimulate more extracellular enzyme production to break soil organic matter (SOM) to access SOM-C [34]. The simultaneously released SOM-N and SOM-C might provide available substrates for subsequent N_2_O emission [35]. However, it is hardly to determine which N_2_O pathway contributing most to the process without the SP signatures of heterotrophic nitrification. The only certainty is that N_2_O is mainly derived from SOM-N, because its δ^15^N^bulk^ showed an opposite increasing pattern with more NH_4_NO_3_ addition relative to agricultural soil.

Compared with the application rates of N fertilizer in the literature (ranging from 20 to 1600 mg N kg^−1^ soil), our added NH_4_NO_3_ rates were not high, but they still significantly altered the N_2_O isotopocule signatures and the N_2_O source contributions in both soils. N fertilizer can amplify N_2_O flux to reach the detection limits for accurate isotopocule measurements, but its impact on soil N_2_O production processes cannot be ignored. In our study, the contributions of autotrophic nitrification and fungal denitrification remarkably increased when only 20 mg N kg^−1^ soil NH_4_NO_3_ was applied in agricultural soil. In forest soil, added NH_4_NO_3_ acted as an external stimulus to produce more N_2_O from SOM, although its exact contribution shift was temporarily incalculable. In the few studies in the literature that used an isotopocule method to investigate forest soil N_2_O emission, much higher application rates (500 or 1470 mg N kg^−1^ soil) were applied in soil incubation experiments [15,18]. Such high inputs are apparently larger than most in agricultural or grassland soil. They would disturb natural forest soil ecosystems, but their impacts can vary depending on different soil ecosystems. Therefore, we propose that N fertilizer should be applied according to its real application rate in agricultural soil and should be avoided in forest soil. For those N deposition studies, N fertilizer should be applied strictly according to the real N deposition amount. To enlarge the N_2_O flux, increasing the incubation size of soil may be an appropriate alternative.

## 5. Conclusions

In conclusion, while only two types of soil from an acidic subtropical area were involved in this study, our data suggest that adding NH_4_NO_3_ significantly increased N_2_O production, changed N_2_O isotopocule signatures and altered N_2_O source contributions. The increased N_2_O production in the agricultural soil was predominantly derived from added NH_4_NO_3_, while it mainly came from SOM-N in the forest soil. Overall, the results presented here provide a basis for conducting soil incubation experiments for N_2_O source partition using an isotopocule method. N fertilizer should be applied according to its field application rate in agricultural soil, while it should be avoided or applied based on the N deposition amount in forest soil. As an alternative, amplifying the soil incubation size would help to achieve enough N_2_O flux for isotopocule measurements. Since heterotrophic nitrification is a major N_2_O source in acidic subtropical forest soil, further experiments are needed to elucidate its isotopocule signatures.

## Figures and Tables

**Table 1:** Soil properties of agricultural and forest soil (means ± SD, n = 3).

Soil Type	pH	TN (g N kg^−1^)	SOC (g C kg^−1^)	C/N Ratio	NH_4_-N (mg kg^−1^)	NO_3_-N (mg kg^−1^)
**Agricultural**	4.8 ± 0.1	0.8 ± 0.0	9.5 ± 0.2	11.5 ± 0.6	17.1 ± 1.5	60.7 ± 2.9
**Forest**	4.6 ± 0.2	1.0 ± 0.1	21.2 ± 4.4	21.5 ± 3.1	6.3 ± 1.4	13.8 ± 5.0

**Table 2:** Isotopocule ratios of N_2_O flux with different NH_4_NO_3_ application rates in agricultural soils.

NH_4_NO_3_ Application (mg N kg^−1^ Soil)	N_2_O Flux (μg kg^−1^ d^−1^)	δ^15^N^bulk^ (‰)	δ^18^O (‰)	SP (‰)
0	3.0 ± 0.2a	−26.4 ± 1.8d	39.0 ± 1.1b	24.9 ± 1.3a
20	3.7 ± 0.2b	−32.2 ± 0.9c	37.8 ± 0.2a	26.1 ± 2.8ab
40	4.7 ± 0.3c	−35.2 ± 1.8b	38.2 ± 0.5ab	26.6 ± 0.4ab
80	5.5 ± 0.8d	−39.2 ± 1.0a	38.1 ± 0.4ab	26.4 ± 1.3ab
160	6.0 ± 0.5d	−38.1 ± 1.4a	39.0 ± 0.4bc	28.0 ± 1.1b

Identical letters indicate no significant differences in average values. ± represents standard deviation.

**Table 3:** Isotopocule ratios of N_2_O flux with different NH_4_NO_3_ application rates in forest soils.

NH_4_NO_3_ Application (mg N kg^−1^ Soil)	N_2_O Flux (μg kg^−1^ d^−1^)	δ^15^N^bulk^ (‰)	δ^18^O (‰)	SP (‰)
0	1.8 ± 0.1a	−14.6 ± 0.6a	34.5 ± 0.8a	15.0 ± 1.4a
20	1.9 ± 0.2ac	−14.0 ± 1.2ab	35.5 ± 1.1ab	15.0 ± 2.4a
40	2.0 ± 0.2bc	−12.3 ± 0.5bc	35.8 ± 0.5b	14.3 ± 2.4a
80	2.0 ± 0.2c	−12.0 ± 1.4c	36.5 ± 0.6cd	18.0 ± 1.8b
160	2.0 ± 0.1c	−10.7 ± 0.8c	37.3 ± 0.3d	18.4 ± 1.1b

Identical letters indicate no significant differences in average values. ± represents standard deviation.

**Table 4:** Contributions of different pathways of N_2_O production assuming that N_2_O from different N_2_O pathways mixed before reduction.

Soil Type	NH_4_NO_3_ Application (mg N kg^−1^ soil)	Case 1		Case 2		Case 3		Case 4	
		Contribution to N_2_O Production (%)		Contribution to N_2_O Production (%)		Contribution to N_2_O Production (%)		Contribution to N_2_O Production (%)	
		Bacterial Nitrification	Nitrifier Denitrification	Bacterial Nitrification	Denitrifier Denitrification	Fungal Denitrification	Nitrifier Denitrification	Fungal Denitrification	Denitrifier Denitrification
Agricultural soil	0	59 (5)	41 (6)	70 (6)	30 (5)	54 (7)	46 (5)	61 (8)	39 (6)
	20	61 (6)	39 (3)	76 (6)	24 (5)	73 (6)	27 (5)	85 (8)	15 (4)
	40	73 (7)	27 (5)	83 (8)	17 (4)	77 (8)	23 (6)	93 (7)	7(4)
	80	78 (7)	22 (6)	88 (8)	13 (6)	100	0	100 (6)	0 (6)
	160	67 (4)	33 (4)	70 (5)	30 (8)	55 (7)	45 (7)	65 (8)	35 (7)
Forest soil	0	0	100	29 (5)	71 (7)	0	100	42 (7)	58 (7)
	20	0	100	0	100	0	100	0	100
	40	0	100	0	100	0	100	1 (5)	99 (6)
	80	0	100	10 (5)	90 (8)	0	100	100	0
	160	0	100	12 (6)	88 (8)	0	100	18 (4)	82 (6)

The uncertainties of contributions are shown in brackets.

## Data Availability

There is no supplemental data in this manuscript.
